# Bridging the gap: empowering physiotherapy students to cultivate patient engagement

**DOI:** 10.1186/s12909-025-06955-9

**Published:** 2025-04-01

**Authors:** James Roberts, Bradley Stephen Neal, Adrian Mallows

**Affiliations:** School of Sport, Rehabilitation and Exercise Sciences, University of Essex, Wivenhoe Park, Colchester, Essex CO4 3WA UK

**Keywords:** Education, Co-creation, Patient involvement

## Abstract

This comment article highlights the often-overlooked voices of patients in healthcare and students in education. It advocates for co-creation in physiotherapy education to help students develop the skills and confidence needed to support greater patient involvement in healthcare, as outlined in the NHS long-term plan.

The Lord Darzi report recognises the National Health Service (NHS) as more than a healthcare system, “it is a social movement with people working together to treat sickness and achieve better health.” [1] Supporting greater patient involvement through approaches such as shared decision making and self-management support is valued by patients and clinicians [2] and was outlined as a focus for the NHS in its 2018 long-term plan [3]. Empowering patients to have the knowledge, skills and confidence to be involved in their healthcare decisions has been titled “the blockbuster drug of the century” [4], resulting in better outcomes, experiences, lessening of health inequalities, and reducing avoidable usage of services [5]. 

Resources to facilitate patient involvement have been developed by both the National Institute of Health and Care Excellence and the Personalised Care Institute [6]. Despite these resources being referred to in evidence-based guidelines that are designed to change practice [7], clinical practice remains didactical and patient involvement in their care can be limited [8]. Physiotherapists’ implementation of these approaches is particularly underwhelming in musculoskeletal healthcare [9]. One reason could be the reported seventeen-year time lag from idea to practice, with healthcare professionals described as ‘resistant to change’ [7]. Other barriers in healthcare have been explored, for example clinicians feeling it is their responsibility to act in the best interests of their patients, or that patients can assume a passive role and not want to be involved [8], conversely patients report feeling unable rather than unwilling to speak up or feeling pressure to be compliant [10]. 

Parallels with healthcare can be seen in education, where the push for evidence-informed teaching to support greater student participation is not yet commonplace [11]. Similar to the imbalance of power in healthcare, students often lack agency and voice [12], and the focus of the knowledge and skills to be acquired is influenced by a historical conception of paternalistic teaching [13]. This is particularly important as physiotherapy clinical practice reflects what students are taught during undergraduate training [14]. A hierarchical approach undervalues and underutilises the knowledge and skills that students bring to the classroom; particularly as greater involvement and autonomy can increase student motivation [15]. 

Students working collaboratively as co-creators in their own learning has been of developing interest in recent years. The term co-creation is defined as “meaningful collaboration between students and staff, with students becoming more active participants in the learning process, constructing understanding and resources with academic staff*”* [16]. Active participation has been identified as the preferred physiotherapy learning style [17], and co-creation allows authentic opportunities for meaningful collaboration, supporting students to become stakeholders in their education and co-create teaching content, pedagogical approaches, assessment, and curriculum design [18]. Students involved in co-creation report greater performance, self-directed learning, self-regulation, and improved confidence and engagement [11]. A greater understanding of students’ perspectives can foster closer partnerships and support the delivery of more effective solutions [11]. Moreover, co-creation serves to strengthen these partnerships while offering students opportunities to cultivate essential relationship-building skills. Fundamentally, both healthcare and education are grounded in human connection, and “when people are supported by strong human relationships, change happens.” [19].

The paradigm shift required to facilitate greater patient involvement and help future clinicians may benefit from starting in an educational setting. Developing relationships and active involvement should become a regular experience over and above the taught knowledge to be acquired. Having shared responsibility with lecturers for their learning and greater involvement; sharing their previous experiences to set individual goals and identify actions to achieve required learning outcomes may support more meaningful and effective personalised learning environments. This parallels with approaches required to facilitate greater patient involvement in clinical practice, which include problem solving, goal setting, barrier identification, and action plans. With a reduced emphasis on knowledge acquisition, collaborative relationships with lecturers may offer the greatest simulation to support clinicians in developing the skills required to feel more comfortable with greater patient involvement and reduce the delivery of low-value care. New physiotherapy graduates often lack confidence in their capabilities upon graduation, and this involvement can support the development of self-efficacy in physiotherapeutic competencies [20]. 

Within the recent independent investigation of the NHS in England, patient voices were frequently reported to be unheard and change is required to offer patients greater involvement in their healthcare [1]. As the NHS places greater emphasis on population health and transitions to place-based approaches, allied health professionals are driving innovative community-created solutions through co-production [21]. Future clinicians will not only require the skills to engage individuals but also build equal partnerships with communities to foster trust and confidence in services. At university, by moving away from managing the perceived needs of an educator and recognising student voices, we can support the development of these essential professional capabilities. By shifting paradigms in education away from paternalism and towards empowerment through co-creation, graduates may be enabled to empower the patients of the future. Parallels between education and healthcare should no longer primarily focus on taught knowledge and more on the shared question: “How can I support you in your journey?”

## Data Availability

No datasets were generated or analysed during the current study.

## Abbreviations

NHS: National Health Service
